# Consensus-based guidelines on subtrochanteric femur fractures: Bridging evidence and experience on 11 key clinical dilemmas

**DOI:** 10.1051/sicotj/2025060

**Published:** 2025-12-05

**Authors:** Swapnil Keny, Gaurav Sharma, Murali Poduval, Anjali Tiwari, Vaibhav Bagaria

**Affiliations:** Department of Orthopedic Surgery, Sir H N Reliance Foundation Hospital, Girgaum Mumbai Maharashtra 400004 India

**Keywords:** Subtrochanteric femur fracture, Cephalomedullary nailing, Fracture fixation, Orthopaedic trauma, Expert consensus

## Abstract

*Background*: Subtrochanteric femur fractures present complex biomechanical and biological challenges with considerable variability in management approaches. Despite a structured approach to operative fixation using the intramedullary nail being accepted as the gold standard for most subtrochanteric fractures, a number of high-impact clinical dilemmas lack clarity and consensus on management approaches due to limited high-level clinical and published evidence. *Methodology*: We identified 11 key controversies through a comprehensive literature review of the PubMed, Scopus, and Cochrane databases from 2011 to 2024. Expert input through direct conversations with high-volume trauma surgeons further reinforced the selection of these problem statements. A modified Delphi consensus process was used to engage with 64 experienced Indian orthopedic surgeons. A four-phase methodology was employed, beginning with the pre-definition of 11 key controversies through literature review. PubMed/Scopus/Cochrane: 2000–2024 and expert input. Phase I description: Before initiating the consensus process, a steering committee systematically reviewed existing literature to predefine and shortlist 11 high-impact, unresolved clinical dilemmas. This ensured all subsequent phases of evidence synthesis and voting were focused on these predefined domains. *Results*: Eleven evidence-supported consensus statements were ratified, addressing implant selection, reduction techniques, technical nuances, and complex scenarios. All statements included clinical rationale, consensus strength (10 Strong, 1 Moderate), and evidence level (I–III). Key outputs of the meeting were the formulation of a standardized treatment algorithm and a decision-making framework for ambiguous clinical situations. *Conclusion*: This consensus provides practical, expert-endorsed guidance to resolve recurring controversies in subtrochanteric fracture management. By bridging evidence gaps with collective surgical experience, it aims to standardize care, reduce unwarranted variation, and improve patient outcomes.

## Introduction

Subtrochanteric femur fractures remain one of the most challenging injuries in orthopedic trauma surgery [1–3]. Located between the lesser trochanter and 5 cm distal to it, this region is subject to immense mechanical stress, dense cortical bone, and poor vascularity, all of which contribute to a high risk of malunion, nonunion, and fixation failure [1, 3, 4]. While significant advancements have been made in implant technology and surgical technique, substantial variation exists in how these fractures are approached, particularly in complex or borderline scenarios [2, 5–7].

**Figure 1:**
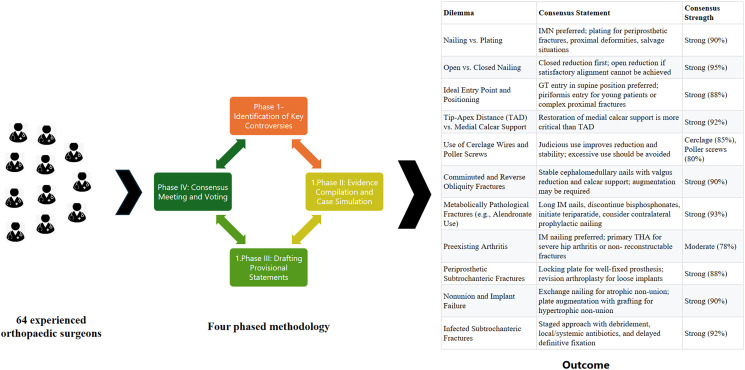
Consensus development methodology; schematic representation of the modified Delphi process used to develop subtrochanteric fracture management guidelines.

Despite the widespread adoption of intramedullary nailing (IMN) as the preferred fixation method, several key controversies remain unresolved in both academic literature and surgical practices. These include the choice between IM nailing and plating [8–15], open versus closed reduction techniques [2, 5, 16], the ideal entry point and patient positioning [4, 7, 17], the relative importance of tip-apex distance (TAD) versus medial calcar support [14, 18], and the use of adjuncts like cerclage wires and poller screws. Added to these are more nuanced scenarios, such as fracture patterns with reverse obliquity or comminution, fractures in the setting of metabolic bone disease (e.g., bisphosphonate use), pre-existing hip arthritis, periprosthetic involvement, nonunion, implant failure, and infection [12, 13, 19–22] (Figure 1).

Literature provides data across a range of study designs and quality levels, yet in many instances, there is insufficient, high-level evidence to guide definitive decision-making [8, 23, 24]. On the other hand, clinical expertise and long-term experience have generated patterns of best practices that often vary across institutions and geographies [7, 10, 20, 21]. This divergence between evidence and eminence often leaves young surgeons, and even experienced practitioners, in a dilemma about the optimal approach to complex subtrochanteric fracture cases [6, 22, 25].

In this context, a national consensus meeting was convened to navigate a middle path between rigid evidence-based protocols and highly individualized surgeon-based decisions. The aim was to bring together both the scientific literature and pragmatic clinical experience through a structured, transparent, and democratic process of discussion, reflection, and agreement [7, 26–28].

This manuscript presents the formal consensus outputs of the Orthopaedic Expert Consensus (OEC) Meeting conducted under the aegis of SICOT India at a tertiary care centre with high-volume trauma experts as participants. The primary objectives were threefold: First, to identify and enumerate the most clinically relevant and recurring controversies in subtrochanteric femur fracture management; second, to synthesize the best available evidence and expert opinion into clear, structured, and actionable consensus statements; and third, to develop broad treatment algorithms and guiding principles that accommodate variability in fracture patterns, patient profiles, and surgical contexts.

Through a modified Delphi approach, we systematically evaluated 11 key areas of controversy and developed consensus statements, each supported by rationale, evidence level, and strength of agreement. We believe this effort will help standardize practice, minimize avoidable variability, and ultimately improve patient outcomes in one of the most technically demanding domains of fracture surgery.

## Methodology

### Study design and objective

This study employed a modified Delphi consensus methodology to address 11 clinically relevant and commonly debated dilemmas in the management of subtrochanteric femur fractures. Through a comprehensive literature review search of the PubMed, Scopus and Cochrane databases from 2011-2024 using keywords “Subtrochanteric femur fracture”, “Deforming forces”, “Varus collapse,” “Cortical bone stress” “Intramedullary nailing vs. Plating” “Open reduction vs. closed reduction,” “Consensus strength” “Delphi consensus,” “Clinical practice guidelines” “Level of Evidence [6, 10]. The objective was to develop practical, evidence-informed, and expert-endorsed consensus guidelines to assist orthopedic surgeons in complex decision-making where literature evidence is limited, inconclusive, or evolving.

### Panel composition

A multidisciplinary panel of 64 orthopedic experts from across India was convened, comprising senior faculty and department chairs from tertiary care institutions specializing in complex trauma management; trauma and arthroplasty surgeons maintaining high-volume practices in complex orthopedic trauma and limb reconstruction; and members of national orthopedic societies as well as editorial boards of peer-reviewed journals. All participants possessed a minimum of 10 years of independent surgical experience in managing complex femur fractures. The recruitment of the steering committee happened through an open invitation to the orthopedic surgeons who performed more than 20 subtrochanteric fracture surgeries a year.

### Process overview

**Figure 2:**
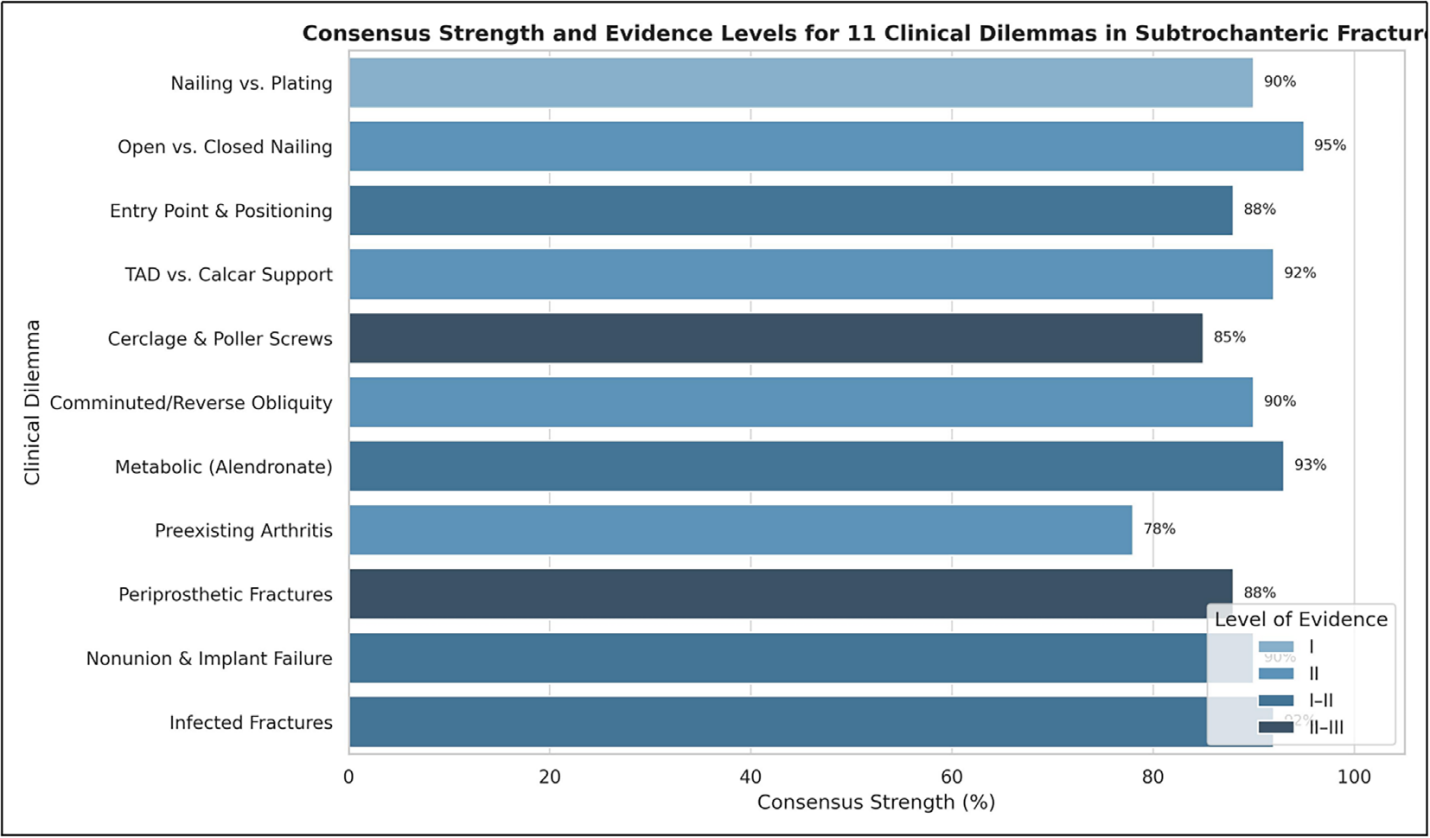
Level of consensus and evidence for the 11 key clinical dilemmas.

The consensus process was carried out in four phases over a 3-month period (Dec 2024 to Feb 2025) (Figure 2).

### Phase I: Identification of key controversies

**Figure 3:**
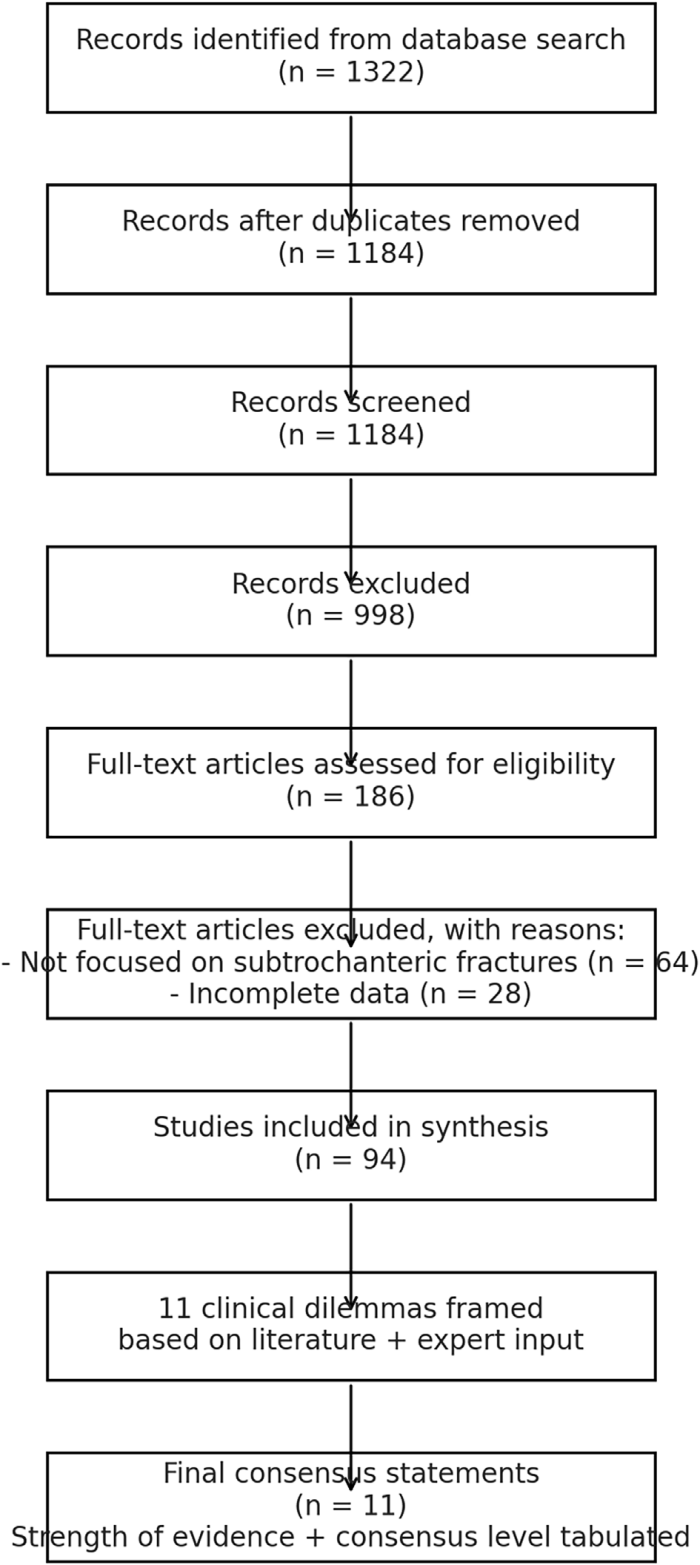
PRISMA guideline for data collection.

A steering committee systematically reviewed existing literature, clinical audit data, and preliminary panelist inputs to identify and shortlist 11 high-impact, unresolved clinical dilemmas in subtrochanteric fracture management (Figure 3). Each dilemma was subsequently refined into a focused clinical question directly applicable to day-to-day orthopedic surgical decision-making. The steering committee comprised the national representative of SICOT from India, the chair of the research member education committee of SICOT, three other senior orthopedic consultants, including a director and a mentor of the tertiary care orthopedic institute.

### Phase II: Evidence compilation and case simulation

For each clinical dilemma, the steering committee conducted a structured literature review adhering to PRISMA guidelines and searched the PubMed, Scopus, and Cochrane databases (2000–2024). Where evidence gaps persisted, simulated clinical cases were developed and presented to panelists to facilitate focused expert deliberation.

### Phase III: Drafting provisional statements

For each clinical dilemma, a working subgroup synthesized literature findings and case discussions to draft a structured provisional consensus statement. These statements systematically included:

A primary clinical recommendation,
Supporting rationale grounded in biomechanical, clinical, or pathophysiological principles, and
The level of supporting evidence.

### Phase IV: Consensus meeting

A dedicated in-person consensus meeting was convened, where each provisional statement underwent rigorous evaluation. Statements were first presented with supporting evidence, followed by a moderated open discussion among panellists. Revisions were implemented iteratively to incorporate expert feedback. Final anonymous voting was conducted using a 3-point Likert scale (Agree/Neutral–Need further evidence/Disagree). Consensus strength was defined as: Strong (≥85% agreement), Moderate (65–84% agreement), or Low (<65% agreement; excluded from formal recommendations).

### Data analysis and validation

Voting responses were analyzed using descriptive statistics. Each consensus statement underwent dual validation: Assignment of both a Level of Evidence and Strength of Consensus. Statements failing to achieve ≥65% agreement were either excluded or designated as priority areas requiring further research.

### Assignment of level of evidence

The Level of Evidence (LOE) for each consensus statement was assigned using the Oxford Centre for Evidence-Based Medicine (OCEBM) 2011 Levels of Evidence framework [29]. This system was chosen for its widespread acceptance and detailed criteria for therapeutic studies. The levels are defined as follows:

**Level I:** Evidence from a systematic review of randomized controlled trials (RCTs) or from high-quality individual RCTs with a narrow confidence interval.
**Level II:** Evidence from prospective cohort studies, lower-quality RCTs, or systematic reviews of these studies.
**Level III:** Evidence from case-control studies, retrospective cohort studies, or systematic reviews of such studies.
**Level IV:** Evidence from case-series or poor-quality cohort and case-control studies.
**Level V:** Evidence from expert opinion without explicit critical appraisal, based on physiology, bench research, or first principles.

The LOE for each statement was determined based on the highest quality evidence available to support the recommendation. For example, a statement primarily supported by a single high-quality RCT would be graded as Level I, whereas a statement derived from a synthesis of retrospective cohort studies and expert biomechanical rationale would be graded as Level III.

### Ethical considerations

As this consensus study involved only expert opinions, without patient data collection or clinical interventions, it qualified for exemption from institutional ethical review per ICMJE guidelines. All 64 participating surgeons provided informed consent for authorship and publication of the consensus outputs.

### Outcome of the process

The final output comprises 11 structured consensus statements, each accompanied by: [1] a clinical rationale grounded in biomechanical, biological, or outcome-based principles; [2] the strength of consensus [categorized as Strong (≥85%), Moderate (65–84%), or Low (<65%)]; [3] the level of supporting evidence (I–V per Oxford CEBM criteria); and [4] key literature references. This document aims to resolve ambiguity in subtrochanteric fracture management by providing practical clinical guidance while promoting standardized decision-making across orthopedic trauma practice settings.

## Results

This consensus process yielded 11 definitive statements that provide comprehensive guidance on the management of subtrochanteric femur fractures, each supported by varying levels of evidence and expert agreement. The recommendations address fundamental aspects from implant selection to management of complex scenarios, with consensus strength categorized as Strong (≥85% agreement) or Moderate (65–84% agreement).

1. Nailing vs. plating

- **Dilemma:** Is intramedullary nailing always preferable, or are there scenarios where plating is better suited?
- **Consensus statement:** *Intramedullary nailing (IMN) is the preferred treatment for most subtrochanteric fractures. Plating may be considered in select cases, such as periprosthetic fractures, proximal deformities, or salvage situations.*
- **Level of evidence:** I
- **Consensus strength: Strong (90%)**
- **Justification:** IMN offers biomechanical and functional advantages; plating is useful in cases where nailing is not feasible or has failed.

2. Open vs. closed nailing

- **Dilemma:** Should anatomical reduction always be attempted closed, or is open reduction acceptable?
- **Consensus statement:** *Closed reduction should be attempted first. Open reduction is justified when satisfactory alignment cannot be achieved or in the presence of comminution requiring cerclage.*
- **Level of evidence:** II
- **Consensus strength: Strong (95%)**
- **Justification:** While preserving biology is ideal, malalignment has worse long-term outcomes.

3. Ideal entry point and positioning

- **Dilemma:** Greater trochanter vs. piriformis fossa entry? Supine vs. lateral position?
- **Consensus statement:** *Greater trochanteric entry in supine position on a traction table is preferred. Piriformis entry and lateral position may be used in young patients or complex proximal fractures.*
- **Level of evidence:** I–II
- **Consensus strength: Strong (88%)**
- **Justification:** GT entry is more accessible and safer; piriformis entry may provide superior alignment in select patients.

4. Tip-apex distance (TAD) vs. medial calcar support

- **Dilemma:** Which is more critical for preventing implant failure?
- **Consensus statement:** *Restoration of medial calcar support is more critical than achieving a specific TAD in subtrochanteric fractures. TAD is relevant only in fractures with intertrochanteric extension.*
- **Level of evidence:** II
- **Consensus strength: Strong (92%)**
- **Justification:** Calcar integrity directly affects mechanical stability and union; TAD is more relevant to intertrochanteric patterns.

5. Use of cerclage wires and poller screws

- **Dilemma:** Do these aids compromise biology or enhance mechanical stability?
- **Consensus statement:** *Cerclage wires and poller screws improve reduction and construct stability when used judiciously. Excessive use should be avoided to preserve biology.*
- **Level of evidence:** II–III
- **Consensus strength:** Cerclage (85%), Poller screws (80%)
- **Justification:** Supportive in comminuted or metaphyseal fractures; to be used as adjuncts, not substitutes.

6. Comminuted and reverse obliquity fractures

- **Dilemma:** Are special techniques needed for these unstable patterns?
- **Consensus statement:** *Comminuted and reverse obliquity fractures should be treated with stable cephalomedullary nails. Valgus reduction and calcar support are critical. Augmentation may be required.*
- **Level of evidence:** II
- **Consensus strength: Strong (90%)**
- **Justification:** These patterns are at high risk for varus collapse and implant failure.

7. Metabolically pathological fractures (e.g., alendronate use)

- **Dilemma:** Do these require a different surgical and medical approach?
- **Consensus statement:** *Long IM nails are recommended. Bisphosphonates should be discontinued. Teriparatide and metabolic optimization are advised. Consider prophylactic nailing of the contralateral femur.*
- **Level of evidence:** I–II
- **Consensus strength: Strong (93%)**
- **Justification:** Suppressed bone turnover delays healing; systemic optimization is essential.

8. Preexisting arthritis

- **Dilemma:** Should primary arthroplasty be considered over fixation in arthritic patients?
- **Consensus statement:** *IM nailing is preferred in most cases. Primary THA may be considered in patients with severe hip arthritis or non-reconstructable proximal fractures.*
- **Level of evidence:** II
- **Consensus strength: Moderate (78%)**
- **Justification:** Balancing between preserving the joint vs. avoiding two surgeries.

9. Periprosthetic subtrochanteric fractures

- **Dilemma:** Fixation or revision arthroplasty?
- **Consensus statement:** *If the prosthesis is well-fixed, locking plate fixation is preferred. Revision arthroplasty is indicated for loose implants.*
- **Level of evidence:** II–III
- **Consensus strength: Strong (88%)**
- **Justification:** Implant stability and bone quality determine the strategy.

10. Nonunion and implant failure

- **Dilemma:** Exchange nailing, plating, or combined constructs?
- **Consensus statement:** *Exchange nailing is preferred for atrophic nonunion; plate augmentation with grafting for hypertrophic nonunion. Dual constructs are indicated in complex failures or bone loss.*
- **Level of evidence:** I–II
- **Consensus strength: Strong (90%)**
- **Justification:** Biomechanical and biological failure must be addressed individually.

11. Infected subtrochanteric fractures

- **Dilemma:** Single-stage vs. staged protocol?
- **Consensus statement:** *A staged approach with debridement, local/systemic antibiotics, and delayed definitive fixation is recommended. Local antibiotic carriers enhance infection control.*
- **Level of evidence:** I–II
- **Consensus strength: Strong (92%)**
- **Justification:** Infection control must precede stability; a staged approach reduces reinfection.

## Discussion

In the field of orthopedic trauma, particularly in complex fractures such as those involving the subtrochanteric region of the femur, clinical decisions often lie in the grey zone between high-level evidence and deep clinical experience. Despite advancements in implant design and fixation techniques, many questions remain unanswered by randomized trials or meta-analyses, compelling surgeons to rely on evidence-based patterns or anecdotal practices. Recognizing this, our consensus initiative aimed to bridge the gap between “what is known” and “what is done” by combining the strength of published evidence with the depth of surgical experience. This approach is not without precedent; similar methods have been employed in orthopedics, notably in the Fragility Fracture Network’s consensus statements and AO Foundation task forces, both of which sought to codify best practices when empirical data alone were insufficient.

Our consensus initiative shares its core objective with established efforts by the Fragility Fracture Network (FFN) and AO Foundation to standardize care and improve outcomes in complex fracture management. However, key differences in scope, methodology, and focus define its unique contribution. While the FFN guidelines offer a comprehensive, system-wide approach to the entire patient journey with fragility fractures, often emphasizing medical optimization and multidisciplinary care [27], our work provides a highly focused, technically granular deep-dive into the specific surgical dilemmas of subtrochanteric femur fractures. Similarly, the AO Foundation principles provide invaluable general frameworks for fracture management and stability [28]. Our consensus builds upon these principles by translating them into explicit, voted-upon recommendations for a specific, high-complication fracture region where literature is often ambiguous. A primary distinction is our use of a structured, multi-round modified Delphi process specifically engaging a large cohort (*n* = 64) of high-volume trauma surgeons within the Indian healthcare context, which encompasses a wide spectrum of resource settings and patient phenotypes. This process was designed to address nuanced technical controversies (e.g., the prioritization of medial calcar support over a specific Tip-Apex Distance) that are often beyond the scope of broader guidelines. Therefore, this document serves as a practical, technically detailed complement to these established frameworks, offering specific guidance for the operating surgeon facing complex intraoperative decisions in subtrochanteric femur fractures.

The first pair of controversies, nailing versus plating, and open versus closed reduction, addresses fundamental questions of surgical philosophy. The panel strongly favored intramedullary nailing as the standard for most subtrochanteric fractures, consistent with biomechanical principles and multiple high-level studies showing its superiority in terms of load-sharing and early mobilization [1–3, 8–10, 15, 30]. However, the consensus was careful to preserve space for plating in specific cases, such as periprosthetic fractures or severe proximal deformities. On the topic of reduction technique, a pragmatic middle path emerged: While closed reduction was favored for its biological advantages, open techniques were endorsed when necessary to achieve anatomical alignment, especially in complex or comminuted patterns [2, 5, 7, 9, 23].

The next thematic pair entry point/positioning and TAD versus medial calcar support—highlighted technical nuances that significantly impact outcomes. The consensus acknowledged greater trochanteric entry in the supine position as the most practical and widely applicable technique [4, 7, 17], while recognizing the piriformis fossa approach as valuable in young or anatomically challenging patients [19–21]. On the reduction front, restoration of the medial calcar was deemed more important than achieving a numeric target for Tip-Apex Distance (TAD), particularly in subtrochanteric patterns where biomechanics trump radiographic metrics. This reflects a shift in thinking from checklist-based fixation to strategic, fracture-specific alignment goals [8, 31].

The discussion around cerclage wires and poller screws, as well as reverse obliquity and comminuted fractures, revealed a consensus leaning toward judicious mechanical augmentation. While traditional teaching discouraged cerclage for fear of disrupting blood supply, recent literature and panel experience have shown that limited, strategic cerclage can greatly aid in achieving reduction without compromising healing [31]. Similarly, poller screws were recognized as essential tools for guiding nail trajectory in widened metaphyseal canals. In highly unstable patterns such as reverse obliquity, there was strong agreement on the need for valgus alignment, calcar restoration, and supplemental fixation as necessary, underscoring that not all nailing is equal, and that technique remains as critical as implant choice.

A more biological lens was required when addressing fractures in the setting of metabolic bone disease (e.g., bisphosphonate-related atypical femoral fractures) and preexisting arthritis. The consensus supported the discontinuation of bisphosphonates and initiation of anabolic agents like teriparatide in such cases, alongside long IM nails and close surveillance for contralateral stress reactions [14, 32]. In patients with hip or knee arthritis, a nuanced approach was adopted: While nailing remains the first-line option, primary arthroplasty was deemed appropriate in select cases with severe joint disease or non-reconstructable fractures. This reflects the panel’s commitment to functional restoration over radiographic perfection.

Finally, the approach to periprosthetic fractures and infected subtrochanteric fractures showcased the panel’s adaptability to complex scenarios [10, 13, 18]. In the former, implant stability was the primary determinant: If well-fixed, locking plate fixation sufficed; if loose, revision arthroplasty was advised. For infected fractures, a staged protocol involving thorough debridement, local antibiotic therapy, and delayed fixation was endorsed, aligning with the principles of chronic osteomyelitis management. The panel strongly discouraged attempts at one-stage fixation in the presence of active infection, highlighting the importance of respecting biology in addition to engineering [12, 13, 26, 32, 33].

Our consensus strongly recommended intramedullary nailing (IMN) as the gold standard for most acute subtrochanteric fractures. This aligns with a large body of literature demonstrating the biomechanical superiority of a load-sharing implant in this high-stress region [1, 3, 5]. However, the existing evidence has a gap: It offers little guidance for borderline cases where patient anatomy or fracture pattern might make plating a viable option (e.g., extremely narrow medullary canals, associated proximal femoral deformity). Our consensus bridges this gap by explicitly defining specific scenarios (e.g., periprosthetic fractures above a stable stem, severe ipsilateral coxa vara) where plating remains a legitimate and preferred choice, providing much-needed nuance to a broadly accepted principle. One of the borderline consensus was on the subject of pre-existing arthritis, where the opinion was divided, particularly because of the duality of skills required as trauma and arthroplasty surgeon.

This new structure transforms the Discussion on the controversies in subtrochanteric fractures from a general commentary into a definitive consensus-driven reference. We hope that this will allow readers to quickly navigate to their topic of interest and understand precisely what new guidance this consensus provides. It also powerfully underscores the necessity and contribution of this work by meticulously documenting the evidence gaps that motivated it.

This consensus document, while comprehensive and rigorously developed, is not without its limitations. The recommendations are inherently shaped by the clinical experiences and perspectives of the participating experts, which, although diverse, may not fully represent global practices or resource settings. Furthermore, several of the controversies addressed lack support from high-level randomized controlled trials, and thus, the consensus statements often rely on lower levels of evidence supplemented by expert judgment. The heterogeneity in implant availability, surgical training, and institutional protocols across regions may also affect the direct applicability of certain recommendations. Additionally, these consensus statements have not yet undergone prospective validation, and as orthopedic science evolves, some of the guidance provided here may require refinement. Despite these limitations, the consensus aims to offer a practical and thoughtful framework for decision-making in an area of orthopedic trauma that remains complex and variably treated.

## Conclusion

Subtrochanteric femur fractures remain one of the most technically demanding injuries to manage in orthopedic trauma. The inherent anatomical, mechanical, and biological challenges are further compounded by clinical dilemmas that often lie in the grey zone between available evidence and surgical judgment. This consensus document, generated through a systematic Delphi process involving 64 experienced orthopedic surgeons, aims to provide a pragmatic framework that balances evidence with eminence.

By addressing 11 key clinical controversies from implant selection and reduction techniques to managing infections, metabolic disease, and periprosthetic fractures, this initiative offers clear, consensus-driven statements with corresponding levels of evidence and strength of agreement. We hope that these guidelines will serve as a useful reference for orthopedic surgeons worldwide, promoting uniformity in decision-making, improved patient outcomes, and a foundation for future prospective studies.

As orthopedic trauma continues to evolve, such collaborative efforts are essential to harmonize care strategies, especially in areas where empirical data alone may not suffice.

## Data Availability

The research data associated with this article are included within the article.
